# The mediating role of emotional intelligence on nursing students’ coping strategies and anxiety during the COVID-19 pandemic

**DOI:** 10.1371/journal.pone.0300057

**Published:** 2024-04-09

**Authors:** Dina Masha’al, Mohammad Rababa, Audai Hayajneh, Ghada Shahrour

**Affiliations:** 1 Adult Health Nursing Department, Faculty of Nursing/ WHO Collaborating Center, Jordan University of Science and Technology, Ar Ramath, Irbid, Jordan; 2 Comunity Health Nursing Department, Faculty of Nursing/ WHO Collaborating Center, Jordan University of Science and Technology, Ar Ramath, Irbid, Jordan; University of Verona, ITALY

## Abstract

Anxiety among nursing students documented during the COVID-19 pandemic reflected their fear of contracting infections, adhering to the mandatory use of masks in public, engaging in the new experience of distance learning, having financial problems, and so on. The purpose of this study was to examine the mediating role of emotional intelligence (EI) on nursing students’ coping strategies and anxiety during the pandemic. This cross-sectional correlational study was conducted in a university in Jordan. An online survey was used to obtain data from a sample of 282 nursing students who had returned to on-campus learning during the summer semester of 2019/2020. The survey held four parts: (a) questions about sociodemographics, (b) the General Anxiety Disorder-7 (GAD-7) scale, (c) the Trait Emotional Intelligence Questionnaire-Short Form (TEIQue-SF), and (d) the Brief-Coping Behavior Questionnaire (Brief-COPE). The results showed that EI had a fully mediating role in the relationship between problem-focused coping strategies and anxiety, and it partially mediated the emotion-focused and avoidant/dysfunctional coping strategies and anxiety relationships. Nursing students who used the problem-focused coping strategies had high levels of EI, and with increasing levels of EI, anxiety levels decreased. Promoting the development of EI among nursing students would enable them to manage their emotions effectively and control their anxiety, particularly in new circumstances such as those that occurred during the COVID 19 pandemic.

## Introduction and background

After 3 months of a government-mandated lockdown in Jordan resulting from the COVID-19 pandemic, nursing students returned to school in the summer semester of 2019/2020 to resume their internships and applied courses in labs, hospitals, and clinics from the previous semester. The students returned to school while the global number of pandemic-related infections and deaths increased dramatically [1].

Nursing students experience high levels of anxiety in normal circumstances [2]. However, fear of contracting infections, dealing with financial difficulties, worrying about their academic progress, experiencing the stress of distance learning, and lacking clinical practice in their clinical courses in the spring semester of 2019/2020 were new sources of stress and anxiety among nursing students upon returning to school [3, 4]. These anxieties would have a negative influence on the students’ academic performance, progress, and quality of life [5, 6].

During the pandemic, rates of anxiety among university students ranged from 25% to more than 87.7% [6–9]. In Jordan, the mean anxiety score of university students on the General Anxiety Disorder-7 (GAD-7) scale during the COVID-19 pandemic was 8.4, which indicated a mild level of anxiety [10]. Another study conducted in Jordan found that more than 70% (*N* = 282) of nursing students who were returning to school after the shutdown experienced mild to severe levels of anxiety [4]. A study conducted in Israel to assess anxiety levels among nursing students who had agreed to work voluntarily in hospitals and the community during the pandemic found that more than 60% of 224 nursing students experienced moderate to severe anxiety levels [6]. Given the high prevalence of anxiety among nursing students during the pandemic, it was crucial to identify how nursing students coped with this anxiety.

The ways that people deal with stressful situations can reduce or amplify the effects of these situations. Effective coping strategies are known to buffer stress and anxiety and facilitate the maintenance of psychological health. Coping may be defined as individuals’ behavioral and cognitive efforts to manage stressful situations that exceed their resources [11]. According to Endler and Parker [12] and Carver [13], coping strategies can be categorized as three types: (a) problem-focused coping, which includes active coping, instrumental support, and planning; (b) emotion-focused coping, which includes acceptance, emotional/social support, humor, positive reframing, and religion; and (c) avoidant/dysfunctional coping, which includes behavioral disengagement, denial, self-distraction, self-blaming, substance use, and venting. Problem-focused coping requires taking actions to modify situations or solve problems, emotion-focused coping requires regulating emotions and/or improving stress management skills, and avoidant/dysfunctional coping requires distancing oneself from stressful situations. Problem-focused coping is considered the most effective type of coping and is linked to lower stress levels, whereas emotion-focused coping and avoidant coping strategies are associated with higher stress levels [14–16].

According to Shikai et al. [17] and Ni et al. [18], problem-focused coping is the most common type of coping strategy among nursing students. However, during the pandemic, most nursing students failed to use problem-focused coping. According to Masha’al et al. [4], most nursing students used avoidant/dysfunctional coping strategies to cope with anxiety. Huang et al. [19] found that during the pandemic, nursing students were less willing than hospital nurses to use problem-focused coping to manage their emotional responses related to COVID-19, which included anxiety, fear, sadness, and anger. Salman et al. [20] found that most Pakistani university students adopted religious/spiritual and acceptance strategies to cope not only with the effects of the pandemic on their daily lives but also their fear of the rapid spread of the virus. Coping strategies have a relationship with psychological problems and health, and emotional intelligence (EI) also has recently been found to correlate with them.

EI refers to the ability of individuals to read their own emotions and the emotions of others; name the different emotions; and use what they know about these emotions to think, behave, and influence others [21, 22]. EI is considered a predictor of critical thinking [23], academic performance [24–26], and mental health [27–29]. In addition, EI influences people’s ability to cope with new situations and remain optimistic, positive, and self-motivated to achieve goals [24].

EI is a trait with two main dimensions: stress management and adaptability [30].It plays a mediatory role between stress and mental health because it helps individuals to cope with environmental conflicts and improve stress management and task performance [14, 31, 32]. Moroń and Biolik-Moroń [33] found that EI predicted lower levels of intensity of fear, anxiety, and sadness among people in Poland during the first week of COVID-19 lockdowns. Compared to individuals with lower EI, individuals with higher EI were found to be more able to adapt to social pressures and environmental changes [34].

Nursing students with high EI have been found to complain less about psychological and physical problems [14, 32, 35, 36]. Individuals with high EI are able to understand and regulate their own emotions and the emotions of others, which enable them to adopt effective coping strategies. Nursing students and nurses with high EI consider stressors less threatening and choose problem-focused coping rather than emotion-focused and/or avoidant coping strategies [14, 15, 31, 37, 38].

To our knowledge, few researchers have examined the relationship between EI, anxiety, and coping strategies during COVID 19 pandemic among nursing students. Further, this is the first study to investigate the mediating role of EI on coping and anxiety. We hypothesized a mediating role of EI on coping strategies and anxiety in nursing students during the pandemic.

## Method

### Design and participant selection

A convenience sample of 400 undergraduate nursing students at Jordan University of Science and Technology was recruited to participate in the study. A total of 282 students completed the survey, leading to a response rate of 70.5%. The 282 questionnaires were returned with no missing data. The remaining 118 questionnaires were incomplete with more than 50% missingness, and thus they were excluded from the analysis. To investigate whether the missing data is completely at random or not, the multivariate diagnostic test was run for this purpose and the analysis revealed that the missing pattern was completely at random (p > .05). The mean of the non-missing items was imputed for the missing scores and all analyses were run with and without the imputed data, showing no significance difference.

All nursing students who were above 18 years old and who had returned to campus in the summer semester of 2019/2020 were included. Students who had taken the summer semester of 2019/2020 off were excluded. Nursing programs in Jordan grant a bachelor’s degree to students attending a total of 134 credit hours, including more than 1800 actual hours in clinical settings divided into 4 academic years.

### Data collection

An online survey using Google Forms was used for data collection. After approval was obtained from the Institutional Review Board (IRB) at Jordan University of Science and Technology, the survey link was sent to students via email. The purpose, procedures, and outcomes of the study were explained on the front page of the survey. The participants also were assured that their identities would remain anonymous, that their participation was voluntary, and that they could quit the survey at any time without consequences. Potential participants were asked to check the “Agree” box if they were willing to participate in the study. This was informed consent to participate in the study. They also were asked to click the “Submit” button at the end of the survey to return it to the researchers. The researchers’ contact information was provided in case of any questions or concerns. Data were collected 2 weeks after the beginning of the summer semester, and the survey link was open for 1 week.

The survey had four parts. The first part held questions about the sociodemographic characteristics of the participants, including age; gender; academic year; stability of financial status (unstable financial status was used to refer to cases where severe changes to family income had occurred during the pandemic); commitment to infection prevention measures (gloves, masks, hand hygiene, etc.); previous infection with COVID-19; and fear of becoming infected after returning to campus. The second part of the survey consisted of the General Anxiety Disorder-7 (GAD-7) scale [39], which was used to assess the students’ levels of anxiety in the 2 weeks preceding data collection (data were collected 2 weeks after the students had returned to campus). The GAD-7 scale comprises seven items that describe the core symptoms of anxiety. The items are scored on a 4-point Likert scale with responses of 0 (*not at all*), 1 (*several days*), 2 (*more than half the days*), and 3 (*almost every day*) [40]. The total possible score ranged from 0 to 21, with a score between 0 and 4 indicating no anxiety, 5 and 9 indicating mild anxiety, 10 and 14 indicating moderate anxiety, and greater than 15 indicating severe anxiety. In the study conducted by Spitzer et al. [39], the scale had good internal inconsistency (Cronbach’s alpha = .92). The third part of the survey held the Trait Emotional Intelligence Questionnaire-Short Form (TEIQue-SF), which was used to measure global trait EI. The questionnaire has two items from each of the 15 subscales of the original TEIQue, with a total of 30 items. Each item is scored on a 7-point Likert scale of responses ranging from 1 (*completely disagree*) to 7 (*completely agree*). The total possible score average of global trait EI ranges from 1 to 7. Higher scores indicate a higher level of trait EI. The instrument assesses four trait EI factors: Well-being (six items), Self-control (six items), Emotionality (eight items), and Sociability (six items). A Cronbach’s alpha of 0.767 has been reported for the questionnaire [41]. The fourth part of the survey held the Brief-Coping Behavior Questionnaire (Brief-COPE), which was used to assess the nursing students’ coping skills. This questionnaire is an abbreviated version of the COPE inventory developed by Carver et al. [42]. The Brief-COPE has 28 items divided between 14 factors of two items each. The items are scored on a 4-point Likert scale of responses ranging from 1 (*I haven’t been doing this at all*) to 4 (*I’ve been doing this a lot*). An internal consistency of 0.83 has been reported for the questionnaire [13]. Coping strategies in the Brief-COPE are divided into problem-focused, emotion-focused, and avoidant/dysfunctional coping strategies. Problem-focused coping includes active coping, instrumental support, and planning, and emotion-focused coping includes acceptance, emotional/social support, humor, positive reframing, and religion. Finally, avoidant/dysfunctional coping includes behavioral disengagement, denial, self-distraction, self-blaming, substance use, and venting [13]. Higher scores indicate respondents’ ways of coping with stressors. The survey was administered in English, the language of instruction in nursing schools in Jordan.

### Data analysis

Data were analyzed using SPSS v.26. Descriptive statistics were used to describe the study sample, anxiety levels, EI levels, and coping strategies. A person’s correlation was computed to identify the relationship between the study main variables. A mediation analysis using PROCESS Macro v.4.2 was carried out to test whether the relationship between coping strategies and anxiety was meditated by EI. The type of coping strategy was treated as the independent variable, with anxiety being the dependent variable and EI the mediator. Variables were tested for multicollinearity, linearity of the residuals, independence, homoscedasticity, and normality. All assumptions were satisfactory.

## Results

### The descriptive statistics

The mean age of the participating nursing students was 20.08 years (*SD* = 1.08). Most of the participants were female students (74.1%), 39.4% were in their second academic year, and 77.3% had a stable financial status. Most of the students had not been infected with COVID-19 (98.9%) and were not afraid of becoming infected (69.1%). The mean score for anxiety was 8.06(5.49) indicating mild level of anxiety. The average EI score among the students was 4.59 (*SD* = 0.74). Problem focused coping showed the highest mean score of the coping mechanism 5.77(1.18). Table 1 illustrates the descriptive statistics of the students’ sociodemographic characteristics, total EI scores, EI factor scores, coping strategies and anxiety.

**Table 1:** Descriptive statistics for students’ sociodemographic variables, anxiety, EI, and coping strategies.

Variable	*N (%)*
**Age** Mean = 20.08 (SD = 1.08)	
**Gender**	
Male	73(25.89)
Female	209(74.11)
**Academic year**	
First year	67(23.76)
Second year	111(39.36)
Third year	65(23.05)
Fourth year	39(13.83)
**Financial Status**	
Stable	218(77.30)
Unstable	64(22.70)
**How frequently did you use the infection preventive measures? (**Hand washing, Masks, gloves….**)**	
Never	27(9.57)
Sometimes	155(54.97)
Always	100(35.46)
**Have you infected by COVID-19 virus?**	
Yes	3(1.06)
No	279(98.94)
**Are you afraid of being infected with COVID 19 virus as you are returning to campus?**	
Yes	87(30.85)
No	195(69.15)
**Emotional intelligence**	*M(SD)*
Emotional intelligence (EI)	4.59(0.74)
Well-being	4.99(1.01)
Self-control	4.41(0.90)
Emotionality	4.41(0.88)
Sociability	4.60(.91)
**Anxiety**	8.06(5.49)
**Coping strategies**	
Problem focused	5.77(1.18)
Emotional focused	5.34(1.06)
Avoidant/dysfunctional	4.38(1.18)

### Correlation between study variables

Before running the mediation analysis, we first examined whether there is a significant relationship between the study main variables using Pearson correlations. The strength of the correlation was interpreted using Cohen’s categorization as the following: “small = .1- .3”; “medium = .3-.5”; and “large = .5–1.0.” The emotion-focused coping and avoidant/dysfunctional coping strategies were correlated positively with anxiety (*p* < .01) and negatively with EI. On the other hand, problem focused coping correlated positively with EI (r = .20, *p* < .01), but the relationship was not significant with anxiety (r = -.01, *p* > 05). Table 2 shows the correlation matrix.

**Table 2:** Correlations between variables.

	Correlation coefficient				
Variable	1	2	3	4	5
1.Anxiety	1				
2.Emotional intelligence	-.47*	1			
3.Problem focused coping	-.01	.20*	1		
4.Emotion focused coping	.17*	-.25*	.55*	1	
5.Avoidant/dysfunctional coping	.18*	-.34*	.40*	.59*	1

*Correlation is significant at the level 0.01 (2-tailed)

### Mediating role of EI on the relationship between coping and anxiety

Three mediational analyses were conducted for each subscale of coping (i.e., problem focused, emotional focused, and avoidance coping). The results revealed a significant indirect effect of problem focused coping on anxiety levels through emotional intelligence (B = -.45, 95% Cl = -.78, -.16, *p* < .001). The total and direct effect of problem focused on anxiety were both insignificant (B = -.04, *t* = -.148, *p* = .88) and (B = .41, *t* = 1.44, *p* = .10), respectively. These results showed that EI exerted a full mediational role on the relationship between problem-focused coping and anxiety.

In regard to emotional-focused coping, the total effect of emotional-focused coping was significantly and positively related to students’ anxiety (B = .87, *t* = 2.88, *p* < .01); however, the direct effect between these two variables in the presence of EI as a mediator was insignificant (B = .29, *t* = 1.06, *p* = .29), and EI partially mediated the relationship between emotion-focused coping and students’ anxiety (B = .57, Cl = .32, .87). In summary, EI partially reduced the effect of emotion-focused coping on anxiety score. Similarly, EI partially mediated the relationship between avoidance coping and anxiety. The results showed that the total effect of avoidance coping on anxiety was significant (B = .83, *t* = 3.07, *p* < .01); however, the direct effect revealed insignificant results between avoidance coping and anxiety score in the presence of EI (B = .01, *t* = .05, *p* = .95) with 95% CI of (LL = .57, UL = 1.10). The coefficients among the variables are illustrated in Table 3.

**Table 3:** The mediating effect of EI on coping strategies and anxiety.

	Total effect			Direct effect			Indirect effect			
IV	*B*	*t*	*p*	*B*	*t*	*P*	*B*	*95% CI*		
								*LL*	*UL*	
Problem focused	-.04	.148	.88	.41	1.44	.10	-.45	-.78		-.16
Emotion focused	.87	2.88	.004	.29	1.06	.29	.57	.32		.85
Avoidance	.83	3.07	.002	.01	.052	.95	.82	.57		1.10

## Discussion

Literature up to date have discussed the relationship between EI, anxiety, and coping mechanisms. However, little is known about the mediating role of EI on the relationship between adopted coping strategies and anxiety. To the best of our knowledge, this is the first study to discuss this mediating role among nursing students during a crisis such as COVID-19 pandemic. We hypothesized that the relationship between coping strategies and anxiety will be mediated by EI.

The results of the study demonstrated a positive relationship between anxiety and emotion focused and avoidant/dysfunctional coping strategies. This is an indication that both coping strategies are maladaptive and increase levels of anxiety. Farnia et al. [43] results reported a negative correlation between test anxiety and problem focused and a positive correlation between test anxiety and emotion focused coping. Zhu et al. [44] demonstrated that positive coping correlated negatively with anxiety during COVID 9 among 165 physician and nurse in China. Savitsky et al. [6] found that during COVID 19, nursing students’ anxiety was linked negatively to their self-esteem and positively with mental disengagement. Reducing maladaptive coping behaviours has been shown to reduce anxiety among college students [45]. The nonsignificant negative correlation between problem focused coping and anxiety may be attributed to the full mediation of EI on the relationship between these two variables (i.e., problem-focused coping and anxiety).

Results showed a full mediation of EI on the relationship between focused -problem coping and anxiety, and partial mediation on the relationship between emotion- focused coping and avoidant/dysfunctional coping and anxiety. These results confirm the role of EI as a key factor to reduce levels of anxiety via coping during the pandemic. According to Por et al. [14], nursing students with higher EI are more capable of managing their emotions and experiencing less stress. In the study by Aghajani Inche Kikanloo et al. [27], higher levels of EI were found to have a positive influence on students’ physiological, emotional, and behavioural responses to stress and stressors. Moroń and Biolik-Moroń [33] found that during the COVID-19 pandemic, trait EI played a protective role against the intensity of some emotional states, including anxiety, fear, and sadness.

It is well documented that high levels of EI are associated with the use of Problem- focused coping [15, 38] and this is consistent with our findings which also revealed that. Higher level of EI might also reduce the chance to use emotion-focused coping and avoidant/dysfunctional coping by students (r = -.25, r = -.34, *p* < .05 respectively).However, some studies contradicted our findings as they reported appositive link between EI and dysfunctional coping. For example, Noorbakhsh et al. [46] conducted a study with a sample of 413 nursing students and found a positive relationship between EI and emotion-focused coping. Further, Yousif Ali et al. [47] found that college nursing others’ emotion appraisal and emotion utilization, which are categories of EI in the Brief Emotional Intelligence Scale, were predictors for self-blame. It has been argued that it is difficult to disentangle problem-focused and emotion-focused coping as they typically co-occur and are intertwined. Emotion-focused coping enhances problem-focused coping by removing emotional distress, which facilitates better problem solving. Meanwhile, problem-focused coping enhances emotion-focused coping by resolving the threat of stress, which reduces distressing emotions [48]. It seems the relationship between EI and the use of adaptive and maladaptive coping is complex as other factors besides EI, such as sociodemographic characteristics, personality factors, environmental factors, and types of stressful situations, are involved in the process of adopting coping strategies [48, 49] including dysfunctional coping strategies. Since this is the first study to examine the mediating role of EI on nursing students’ anxiety via their use of coping, it is crucial to further investigate this process taking into account the aforementioned factors and using different mediational methods such structural equation modeling.

### Implications

The findings of this study highlighted the important role emotional intelligence plays in the reduction of nursing students’ anxiety via the coping strategies employed by them amid COVID-19 pandemic. Favorable coping strategies, namely problem-focused coping was fully mediated and emotion-focused and avoidant/dysfunctional coping were partially mediated by students’ EI to reduce their anxiety. This is very important in times of crises such as COVID-19 pandemic where anxiety is heightened by the fear of getting infected by the virus and inflicting loved ones. Prior research emphasized the need for developing interventions to promote individuals’ well-being during the pandemic (e.g., Wang et al., 2020) [50] and some highlighted the protective role of EI on individual’s psychological health amidst COVID-19 (e.g., Sanchez-Ruiz et al., 2021) [51]. Our study also emphasizes the importance of adopting interventions that increase trait EI in the face of crisis as the findings pointed out to the reduction of students’ anxiety through students’ coping strategies. The current study adds to the increasing body of literature the benefits of EI element in alleviating nursing students’ anxiety in times of adversity as this has not been investigated previously. Although COVID-19 is a transitory infectious disease similar to previous infections such as N1H1 flue and Ebola, future evolving epidemics may occur, and thus fortifying our student’s wellbeing against these pandemics is required and one approach to achieve this goal is through improving their emotional intelligence.

### Limitations

The study had some limitations that need to be taken into consideration in the interpretation of the results. The results were based on self-reported data, which may not have reflected the actual levels of anxiety and EI among the participating nursing students. The cross-sectional design of this study also limited the interpretation of the causes and effects of using certain coping strategies. Recruiting participants from only one university in one geographical area limited the generalizability of the study results. The students also might have influenced each other’s responses because no strategies were considered to avoid communication among the students as they completed the online survey. Although excluding students who did not enroll in the summer semester reduced confounding variability in the studied variables, however, future studies need to ensure their inclusion and see whether differences exist between them and those who are enrolled in the academic setting. Our question of students’ frequency of usage of COVID-19 preventative measure did not specify the context of preventative measures application (i.e., whether the usage was in hospital or daily life context). As a result, the findings related to this question should be interpreted cautiously and future research needs to provide contextual information regarding this item. One crucial limitation of this study lies in the utilization of regression analysis to test the mediation model. Mediation analysis using regression does not draw a cause-effect relationship between the study variables as other co-founding variables may play a role in such case. Re-examining the mediation role of EI on the relationship between coping strategies and anxiety using stronger analysis such as structural equation modeling is called for.

## Conclusion

Nursing students who adopted problem-focused coping strategies have had higher levels of EI and lower levels of anxiety. The analysis revealed a mediating role for EI on the relationship between problem-focused coping strategies and anxiety. The results of the study highlighted the importance of EI in the ability of nursing students to deal with stressful situations such as the COVID-19 pandemic. Because EI traits can be acquired, it is proposed that training courses to strengthen and increase the levels of EI among nursing students be developed and implemented. Further, to our knowledge, no studies have been conducted on the mediating role of EI on coping strategies and anxiety, so we recommend that more research to be conducted to find the causal relationship between and among the variables using stronger mediational analysis such as SEM. A longitudinal study evaluating the effect of EI levels on the coping strategies and anxiety of nursing students, especially during a very stressful situation, is recommended.

## Supporting information

S1 Data (XLS)
